# Stochastic Noise and Synchronisation during *Dictyostelium* Aggregation Make cAMP Oscillations Robust

**DOI:** 10.1371/journal.pcbi.0030218

**Published:** 2007-11-09

**Authors:** Jongrae Kim, Pat Heslop-Harrison, Ian Postlethwaite, Declan G Bates

**Affiliations:** 1 Control and Instrumentation Group, Department of Engineering, University of Leicester, Leicester, United Kingdom; 2 Systems Biology Laboratory, University of Leicester, Leicester, United Kingdom; 3 Department of Biology, University of Leicester, Leicester, United Kingdom; University of California San Diego, United States of America

## Abstract

Stable and robust oscillations in the concentration of adenosine 3′, 5′-cyclic monophosphate (cAMP) are observed during the aggregation phase of starvation-induced development in Dictyostelium discoideum. In this paper we use mathematical modelling together with ideas from robust control theory to identify two factors which appear to make crucial contributions to ensuring the robustness of these oscillations. Firstly, we show that stochastic fluctuations in the molecular interactions play an important role in preserving stable oscillations in the face of variations in the kinetics of the intracellular network. Secondly, we show that synchronisation of the aggregating cells through the diffusion of extracellular cAMP is a key factor in ensuring robustness of the oscillatory waves of cAMP observed in *Dictyostelium* cell cultures to cell-to-cell variations. A striking and quite general implication of the results is that the robustness analysis of models of oscillating biomolecular networks (circadian clocks, Ca^2+^ oscillations, etc.) can only be done reliably by using stochastic simulations, even in the case where molecular concentrations are very high.

## Author Summary

The molecular network, which underlies the oscillations in the concentration of adenosine 3′, 5′-cyclic monophosphate (cAMP) during the aggregation phase of starvation-induced development in Dictyostelium discoideum, achieves remarkable levels of robust performance in the face of environmental variations and cellular heterogeneity. However, the reasons for this robustness remain poorly understood. Tools and concepts from the field of control engineering provide powerful methods for uncovering the mechanisms underlying the robustness of these types of biological systems. Using such methods, two important factors contributing to the robustness of cAMP oscillations in *Dictyostelium* are revealed. First, stochastic fluctuations in the molecular interactions of the intracellular network, arising from random or directional noise and biological sources, play an important role in preserving stable oscillations in the face of variations in the kinetics of the network. Second, synchronisation of the aggregating cells through the diffusion of extracellular cAMP appears to be a key factor in ensuring robustness to cell-to-cell variations of the oscillatory waves of cAMP observed in *Dictyostelium* cell cultures. The conclusions have important general implications for the robustness of oscillating biomolecular networks (whether seen at organism, cell, or intracellular levels and including circadian clocks or Ca^2+^ oscillations, etc.), and suggest that such analysis can be conducted more reliably by using models including stochastic simulations, even in the case where molecular concentrations are very high.

## Introduction

Dictyostelium discoideum are social amoebae which normally live in forest soil, where they feed on bacteria [1]. Under conditions of starvation, *Dictyostelium* cells begin a programme of development during which they aggregate to eventually form spores atop a stalk of vacuolated cells. At the beginning of this process the amoebae become chemotactically sensitive to cAMP, and after about six hours they acquire competence to relay cAMP signals. After eight hours, a few pacemaker cells start to emit cAMP periodically. Surrounding cells move toward the cAMP source and relay the cAMP signal to more distant cells. Eventually, the entire population collects into mound-shaped aggregates containing up to 10^5^ cells ([2], p. 4350). The processes involved in cAMP signalling in *Dictyostelium* are mediated by a family of cell surface cAMP receptors (cARs) that act on a specific heterotrimeric G protein to stimulate actin polymerisation, activation of adenylyl and guanylyl cyclases, and a number of other responses [3]. Most of the components of these pathways have mammalian counterparts, and much effort has been devoted in recent years to the study of signal transduction mechanisms in these simple microorganisms, with the eventual aim of improving understanding of defects in these pathways which may lead to disease in humans [4].

In [5], a model was proposed for the network of interacting proteins involved in generating cAMP oscillations during the early development stage of *Dictyostelium*.

The model, which is written as a set of nonlinear ordinary differential equations, exhibits spontaneous cAMP oscillations of the correct period and amplitude, and also reproduces the experimentally observed interactions of the MAP kinase ERK2 and protein kinase PKA with the cAMP oscillations [6]. In addition to accurate reproduction of experimental data for one chosen set of parameter values, model robustness to parameter uncertainty in appropriate subsets of those parameters has been proposed by several authors in recent years as an important criterion for model validity [7,8]. The idea here is simply that the model's dynamics should not be highly sensitive to changes in parameters whose values either cannot be determined accurately, or are known to vary widely in vivo. In [5], the dynamics of the model are claimed to be highly robust when subjected to trial and error variations of one kinetic parameter at a time. More systematic robustness analyses of this model published in [9] and [10], however, revealed an extreme lack of robustness in the model's dynamics to a set of extremely small perturbations in its parameter space. Since the cAMP oscillations observed in vivo are clearly very robust to wide variations in these parameters, this result could be interpreted as casting some doubt on the validity of the model. On the other hand, there is strong experimental evidence to support each of the stages and interconnections in the proposed network, and the “nominal” model's dynamics show an excellent match to the data.

In this paper, we attempt to resolve this apparent paradox by showing how a stochastic representation of the deterministic model proposed in [5], together with the incorporation of synchronisation effects due to the diffusion of extracellular cAMP between aggregating cells, results in an extremely robust model for cAMP signalling in *Dictyostelium*. The effects of stochastic noise in biomolecular networks have been intensively studied from a number of points of view in recent years [11–16]. Efficient ways of calculating the magnitude of noise in biomolecular networks are described in [17,18]. In addition, the ability of noise to generate oscillations and the effect of noise on the resonant frequency are analysed in [19]. Similar synchronisation structures, i.e., coupled oscillators, are found in many biomolecular networks, for example, glycolytic oscillations in yeast cells [20], circadian oscillations [21], etc. Typically, however, analyses of oscillations in such systems are conducted in a deterministic framework [20,21]. A common feature of all such studies is that they emphasise the necessity of taking stochastic noise effects into account only for models of systems involving very low molecular copy numbers. In this paper, we have an example of a situation where it appears that, at least for the purposes of robustness analysis, stochastic noise effects must be taken into account even for very high intracellular molecular concentrations. In addition, most previous studies that have considered the issue of robustness have investigated robustness of the system *to* the effects of stochastic noise, see for example [12]. The possibility of beneficial effects arising from stochastic fluctuations in genetic and biochemical regulatory systems was first proposed in [22]. The results contained in this paper provide strong evidence that stochastic noise is actually an important *source* of robustness for this, and probably many other, oscillatory biological systems.

## Results

### Stochastic Noise Improves the Robustness of cAMP Oscillations in Individual *Dictyostelium* Cells

**Figure 1:**
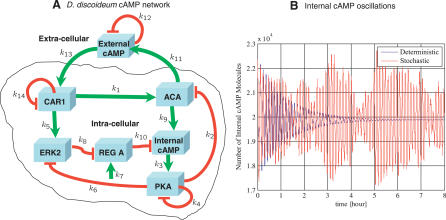
*Dictyostelium* cAMP Oscillations (A) The model of [5] for the network underlying cAMP oscillations in *Dictyostelium*. The nominal parameter values for the model are taken from [6,9] and are given by: *k*_1_ = 2.0 min^−1^, *k*_2_ = 0.9 *μ*M^−1^ min^−1^, *k*_3_ = 2.5 min^−1^, *k*_4_ = 1.5 min^−1^, *k*_5_ = 0.6 min^−1^, *k*_6_ = 0.8 *μ*M^−1^ min^−1^, *k*_7_ = 1.0 *μ*M min^−1^, *k*_8_ = 1.3 *μ*M^−1^ min^−1^ , *k*_9_ = 0.3 min^−1^, *k*_10_ = 0.8 *μ*M^−1^ min^−1^, *k*_11_ = 0.7 min^−1^, *k*_12_ = 4.9 min^−1^, *k*_13_ = 23.0 min^−1^, and *k*_14_ = 4.5 min^−1^. A perturbation of magnitude 2% in the model parameters which causes the oscillations to cease is given by [10]: *k*_1_ = 1.9600, *k*_2_ = 0.8820, *k*_3_ = 2.5500, *k*_4_ = 1.5300, *k*_5_ = 0.5880, *k*_6_ = 0.8160, *k*_7_ = 1.0200, *k*_8_ = 1.2740, *k*_9_ = 0.3060, *k*_10_ = 0.8160, *k*_11_ = 0.6860, *k*_12_ = 4.9980, *k*_13_ = 22.5400, and *k*_14_ = 4.5900. (B) With the above perturbation in the parameter values, the deterministic model stops oscillating. The stochastic model, on the other hand, continues to exhibit stable and robust oscillations.

**Figure 2:**
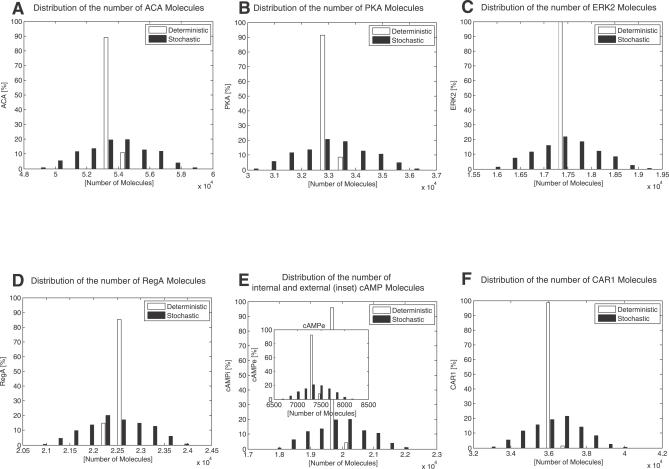
Deterministic and Stochastic Simulations for the Same Worst Case Parameter Combinations Are Performed The numbers of molecules are sampled with a 0.1 s interval for 10 h, and the distribution of each molecular species is compared. To avoid influences from the initial transient response, only the samples obtained after 5 h are considered when plotting the distributions. The inset of (E) is the distribution for the external cAMP. The noise effect is clearly significant in terms of generating oscillations.

The original model for cAMP oscillations given in [5] comprises the set of coupled nonlinear ordinary differential equations shown in Materials and Methods as in Equation 1. The stochastic version of the model is obtained by converting the ordinary differential equations into the corresponding fourteen chemical reactions, Equation 2. The interaction network described by both models is shown in Figure 1A. After external cAMP binds to the cell receptor CAR1, ligand-bound CAR1 activates adenylyl cyclase ACA and the mitogen activated protein kinase ERK2. ACA stimulates the production of cAMP and the cAMP activates the protein kinase PKA. PKA inhibits ACA and ERK2, which form two feedback loops around the internal cAMP. As shown in Figure 1B, a 2% perturbation from the nominal values of the kinetic parameters in the original deterministic model is sufficient to destroy the stability of the oscillation and make the system converge to a steady state in about 6 h [10]. On the other hand, Figure 1B shows that the stochastic model continues to exhibit a stable oscillation for this perturbation to the nominal model parameters. The distributions of the numbers of all molecular species are shown in Figure 2A–2F. For the deterministic model, the numbers of each molecular species are concentrated in a narrow region. On the other hand, for the stochastic case they are relatively widely spread, which shows that the magnitude of noise in the network has a dominant effect in terms of generating oscillations. The critical factor in terms of stochastic noise generating oscillations is the number of molecules in the cell. That is, the magnitude of the noise depends on the square root of the number of molecules. Moreover, the number of molecules is a function of the cell volume as shown in [23]. Hence, unless the cell volume was far larger than that which corresponds to biological reality, the stochastic effects considered here will remain dominant.

**Figure 3:**
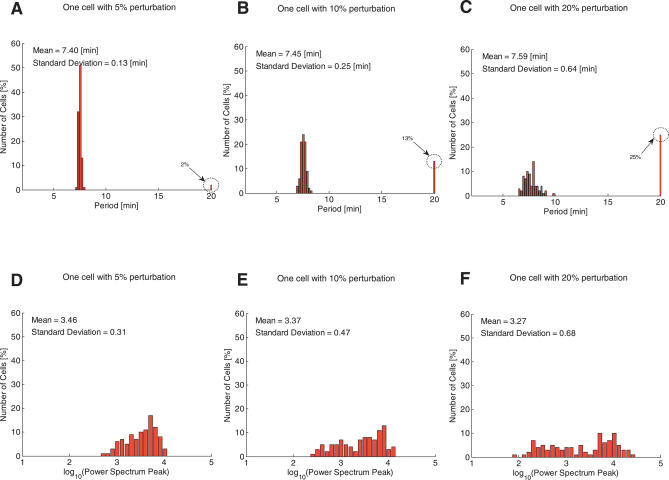
Deterministic Model: Robustness Analysis of the Period and Amplitude of the Internal cAMP Oscillations with Respect to Perturbations in the Model Parameters and Initial Conditions The first row shows the distribution in the period of the deterministic model for one cell with 5%, 10%, and 20% perturbations, and the second row shows the amplitude distribution. The peak bar at 20 min for the period distributions represents the number of cells that are not oscillating. The proportion of cells that are not oscillating increases from 2% to 25% as the size of the perturbation increases. The distributions of the amplitudes also show a similar tendency, i.e., the mean value decreases and the standard deviation increases as the magnitude of the perturbation increases. Each plot is the result of 100 simulations for different random samples of the model parameters, the cell volume, and initial conditions using a uniform distribution about the nominal values.

**Figure 4:**
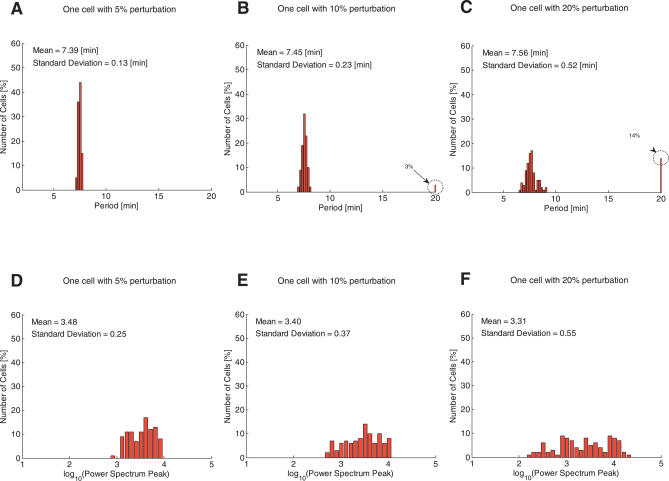
Stochastic Model: Robustness Analysis of the Period and Amplitude of the Internal cAMP Oscillations with Respect to Perturbations in the Model Parameters and Initial Conditions The first row shows the period distribution of the stochastic model for one cell with 5%, 10%, and 20% perturbations, and the second row shows the amplitude distribution. The peak bar at 20 min for the period distributions represents the number of cells that are not oscillating. The proportion of cells that is not oscillating increases from 0% to 14% as the size of the perturbation increases, and is always significantly smaller than the proportion of non-oscillating cells found in the deterministic model. The standard deviations of the amplitudes are also much smaller, for the same magnitude of perturbation, than those seen in the deterministic model. Each plot is the result of 100 simulations for different random samples of the model parameters, the cell volume, and initial conditions using a uniform distribution about the nominal values.

To systematically compare the robustness properties of the two models, we generated 100 random samples of kinetic constants, the cell volume, and initial conditions from uniform distributions around the nominal values for several different uncertainty ranges. The period distributions of the deterministic model for three uncertainty ranges, i.e., 5%, 10%, and 20%, are shown in Figure 3A–3C. The same results for the stochastic model are shown in Figure 4A–4C. In the figures, the peak at the 20 min period denotes the total number of cases where the trajectories converged to some steady state value. Note that the proportion of non-oscillatory trajectories is already 2% for the deterministic model with just a 5% level of uncertainty. On the other hand, for a 5% level of uncertainty in the model parameters, the stochastic model shows perfect robustness, with not a single converging case discovered in the simulations. In fact, for perturbations of up to 20%, a significant majority of cases still displayed stable oscillations, with only 14% converging to a steady state. Similar improvements in the robustness of the amplitude distributions are shown in Figure 3D–3F and Figure 4D–4F. For a 5% level of uncertainty, the variation in the amplitude of the oscillation is much wider for the deterministic model, while for perturbations of up to 10% and 20% its amplitude distribution seems to become almost bimodal. For the stochastic model, on the other hand, the standard deviations of the amplitude for all cases are smaller than that for the deterministic cases.

### Synchronisation of Oscillations in Aggregating *Dictyostelium* Cells Provides Robustness to Cell-to-Cell Variations

One important mechanism, which is missing in the model of [5], is the communication between neighbouring *Dictyostelium* cells through the diffusion of extracellular cAMP. During aggregation, *Dictyostelium* cells not only emit cAMP through the cell wall but also respond to changes in the concentration of the external signal which result from the diffusion of cAMP from large numbers of neighbouring cells. The authors in [24] clarified how cAMP diffusion between neighbouring cells is crucial in achieving the synchronization of the oscillations required to allow aggregation. Interestingly, similar synchronisation mechanisms have been observed in the context of circadian rhythms—the consequences and implications of such mechanisms are discussed in [25].

**Figure 5:**
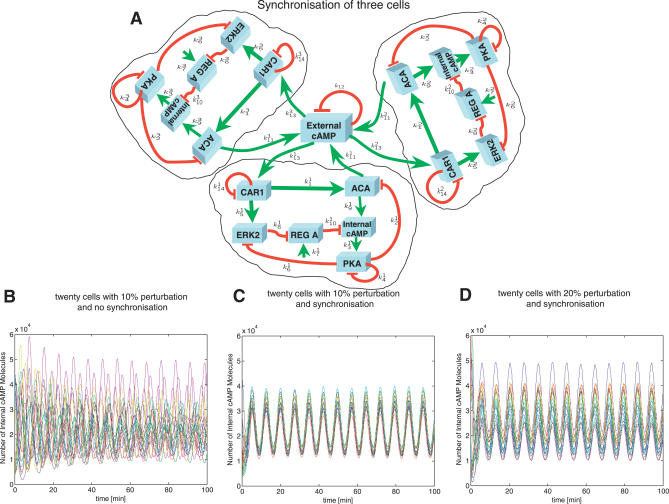
Synchronisation Is Realised through Diffusion of External cAMP (A) Shows the synchronisation mechanism for the case of three interacting *Dictyostelium* cells. Each cell has a different set of kinetic constants. *k^i^_j_* is the *k_j_* in the Laub-Loomis model for the *i*-th cell. (B) For twenty individual cells with no interaction and a 10% level of variation in the initial conditions and the kinetic constants between the cells, the internal cAMP oscillations are completely out of phase with each other. (C) For the extended model incorporating the diffusion mechanism, with the same level of variation between the cells, the oscillations are synchronised in less than 10 min. (D) Even for a 20% level of variation between the cells, the extended model shows highly synchronised oscillations.

To investigate the effect of synchronisation on the robustness of cAMP oscillations in *Dictyostelium*, we extended the stochastic version of the model of [5] to capture the interactions between cells as described in Materials and Methods. Figure 5A shows an example of the extended model for the case of three cells in close proximity to each other. Because each cell is not exactly the same, the kinetic constants and initial conditions are assumed to be different for each individual cell model. As shown in Figure 5B, with just a 10% level of variation among the different cells' kinetic parameters, the cell volume, and initial conditions, the oscillations generated by 20 non-interacting cells will be completely asynchronous with each other after only 10 min. On the other hand, the extended model which allows communication between the cells through the diffusion of cAMP provides synchronised and stable oscillations for variations of up to 20% in the parameters of the individual cells—Figure 5C and 5D. Thus the dynamics of the cAMP oscillations appear to depend strongly on the strength of synchronisation between the individual cells, as well as on the level of cell-to-cell variation. These factors may in fact be the critical mechanisms for developing morphogenetic shapes in *Dictyostelium* development—note that [26] showed that cell-to-cell variations desynchronise the developmental path and argued that they represent the key factor in the development of spiral patterns of cAMP waves during aggregation.

**Figure 6:**
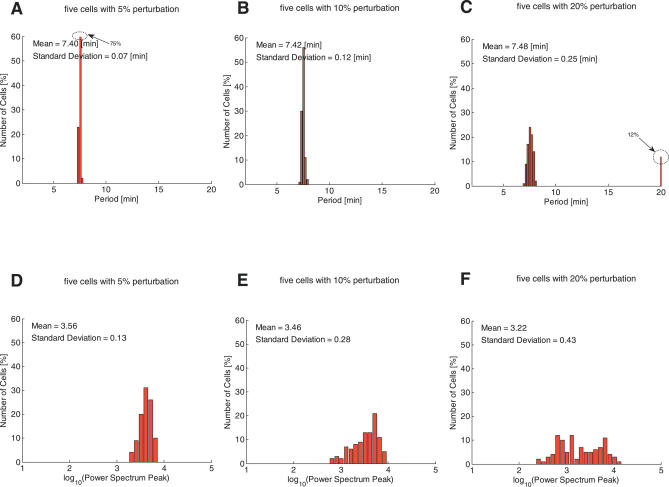
Extended Stochastic Model (Five Cells): Robustness Analysis of the Period and Amplitude of the Internal cAMP Oscillations with Respect to Perturbations in the Model Parameters and Initial Conditions The first row shows the period distribution of the stochastic model for three cells with 5%, 10%, and 20% perturbations, and the second row shows the amplitude distribution. The peak bar at 20 min for the period distributions represents the total number of cells that are not oscillating. The proportion of non-oscillating cells increases from 0% to 12% as the size of the perturbation increases, which is smaller than the proportion seen in either the deterministic or stochastic single cell models. The distributions of the amplitudes show a similar tendency, i.e., the mean decreases and the standard deviation increases as the magnitude of the perturbation increases. Each plot is the result of 100 simulations for different random samples of the model parameters, the cell volume, and initial conditions using a uniform distribution about the nominal values.

**Figure 7:**
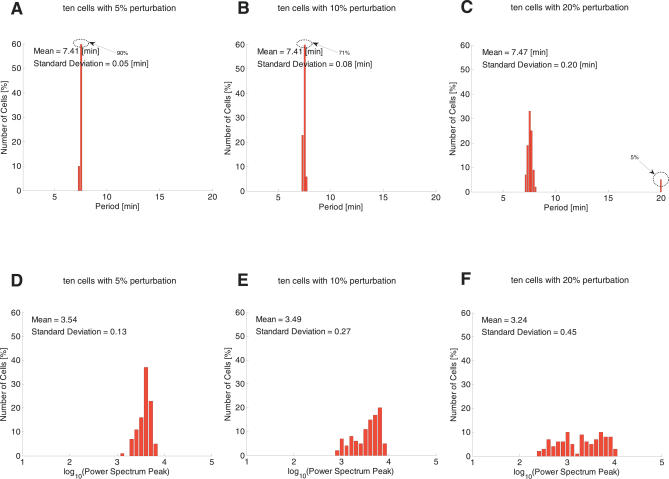
Extended Stochastic Model (Ten Cells): Robustness Analysis of the Period and Amplitude of the Internal cAMP Oscillations with Respect to Perturbations in the Model Parameters and Initial Conditions The first row shows the period distribution of the stochastic model for three cells with 5%, 10%, and 20% perturbations, and the second row shows the amplitude distribution. The peak bar at 20 min for the period distributions represents the total number of cells, which are not oscillating. The proportion of non-oscillating cells increases from 0% to 5% as the size of the perturbation increases, which is much smaller than the proportion seen in all other cases. The distributions of the amplitudes show a similar tendency, i.e., the mean decreases and the standard deviation increases as the magnitude of the perturbation increases. Each plot is the result of 100 simulations for different random samples of the model parameters, the cell volume, and initial conditions using a uniform distribution about the nominal values.

Robustness analysis results for the extended model in the case of five and ten interacting cells are shown in Figures 6 and 7. Figure 6A–6C and Figure 7A–7C show that the variation in the period of the oscillations reduces as the number of synchronised cells in the extended model increases. The proportion of non-oscillating trajectories for the five-cell extended model with a level of variation between the cells of 20% is only 12% of the total. This proportion is further reduced as the number of synchronised cells increases. For the extended model with ten cells, the first non-oscillating cells appear with a 20% level of variation and these make up only 5% of the total. The mean values of the amplitude distributions, shown in Figures 6D–6F and 7D–7F, are more or less similar. However, it may be the case that greater effects on the amplitude distribution are produced for larger numbers of cells.

Note that for computational reasons the number of interacting cells considered in the above analysis was limited to ten. In nature, some 10^5^ *Dictyostelium* cells form aggregates leading to slug formation, and each cell potentially interacts with far more than ten other cells. The stochastic model here suggests how either direct or indirect interactions will lead to even stronger robustness of the cAMP oscillations as well as entrapment and synchronization of additional cells.

## Discussion

As well as resolving an apparent paradox concerning the robustness of a proposed model for cAMP oscillations in *Dictyostelium* cells, the results of this study make some interesting contributions to the “stochastic versus deterministic” modelling and simulation debate in Systems Biology. Generally speaking, the arguments in favour of employing a stochastic framework for the modelling of intracellular dynamics have focused on the case of systems involving small numbers of molecules, where large variabilities in molecular populations favour a stochastic representation. Of course, this immediately raises the question of what exactly is meant by “small numbers”—see [27] for an interesting discussion of this issue. In this paper, however, we have analysed a system in which molecular numbers are very large, but the choice of a deterministic or stochastic representation still makes an enormous difference to the robustness properties of the network model. The implications are clear—when using robustness analysis to check the validity of models for oscillating biomolecular networks, only stochastic models should be used. The reason for this is due to the second major result of the paper—intracellular stochastic noise can constitute an important *source* of robustness for oscillatory biomolecular networks, and therefore must be taken into account when analysing the robustness of any proposed model for such a system. Finally, we showed that biological systems that are composed of networks of individual stochastic oscillators (e.g., aggregating *Dictyostelium* cells) use diffusion and synchronisation to produce wave patterns which are highly robust to variations among the components of the network.

##  Materials and Methods

### The deterministic model.

The deterministic model for cAMP oscillations used in this study is taken from [5] and is given by

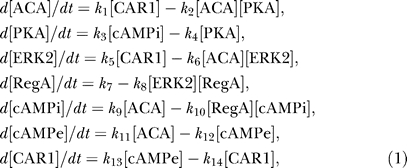
where ACA is adenylyl cyclase, PKA is the protein kinase, ERK2 is the mitogen-activated protein kinase, RegA is the cAMP phosphodiesterase, cAMPi and cAMPe are the internal and the external cAMP concentrations, respectively, and CAR1 is the ligand-bound cell receptor.

### The stochastic model.

To transform the above ordinary differential equations into the corresponding stochastic model, the following fourteen chemical reactions are deduced [28]:

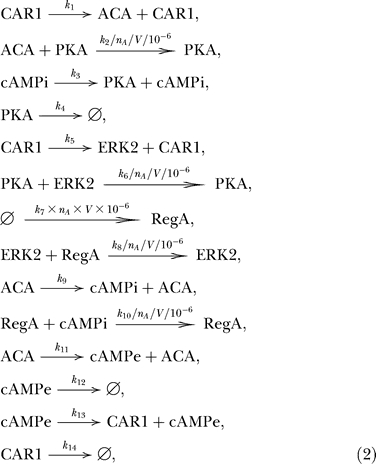
where ∅︀ represents some relatively abundant source of molecules or a non-interacting product, *n*_A_ is Avogadro's number, 6.023 × 10^23^, 10^−6^ is a multiplication factor due to the unit *μ*M, and *V* is the size of the volume where the reactions occur. In our computations, we chose *V* equal to 3.672 × 10^−14^ *l*, to ensure that for the nominal kinetic parameter values the average number of ligand-bound CAR1 molecules corresponds to the average number of CAR1 receptors on the surface of a *Dictyostelium* cell 4 h after the initiation of development, which is around 40,000 [5]. The probability of each reaction occurring is defined by the rate of each reaction. For example, the probabilities during a small length of time, *dt*, that the first and the second reactions occur are given by *k*_1_ × CAR1 and *k*_2_/*n*_A_/*V*/10^−6^ × ACA × PKA, respectively. The probabilities for all the other reactions are defined similarly. Based on these, the chemical master equation is obtained and solved using standard numerical routines [29].

To consider synchronisation between multiple cells, Equation 2 is extended under the assumption that the distance between cells is small enough that diffusion is fast and uniform. In this case, the above reactions for each individual cell just need to be augmented with one reaction that includes the effect of external cAMP emitted by all the other cells. Since the external cAMP diffuses fast and uniformly, the reaction involving *k*_13_ is modified as follows:

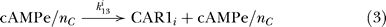
for *i* = 1, 2, …, *n*_c_ − 1, *n*_c_, where cAMPe is the total number of external cAMP molecules emitted by all the interacting cells, *n*_c_ is the total number of cells, *k^i^*_13_ is the *i*-th cell's kinetic constant for binding cAMP to CAR1, and CAR1*_i_* is the *i*-th cell's CAR1 number.

Note that the diffusion constant, *D*, of cAMP is equal to 4.0 × 10^−4^ cm^2^/s [24]. At the stage in the aggregation process considered here, there will be ten cells in a 100 *μ*m × 100 *μ*m rectangular region assuming a density of 10^5^ cells/cm^2^ [26]. The diffusion time is given by *r* ^2^/(6*D*), where *r* is the diffusion distance [30]. Hence, the diffusion time from one corner to the other corner of the rectangular region considered, i.e., the farthest possible distance, is approximately 0.083s. This is orders of magnitude faster than the usual period of cAMP oscillations, which is between 5 min and 10 min. Therefore, the effect of diffusion speed, i.e., the effect of cAMP spatial distributions on cAMP oscillations will be minor during this stage of aggregation. However, if the distance between cells is very large, as could be the case in the early stages of aggregation, then the spatial distribution will have a significant effect, and a corresponding wave of cAMP over the region is observed. On the other hand, if the distance between cells becomes very small, then most of the cAMP molecules will be almost immediately bound to the receptors before diffusion can occur. Indeed, these issues could be proposed as a possible explanation for the qualitative changes in *Dictyostelium* which occur after aggregation.

### Random sampling for Monte-Carlo simulations.

To ensure a consistent procedure for checking the robustness of both the deterministic and stochastic models, the Monte-Carlo simulation technique is used. The kinetic constants are sampled uniformly from the following:

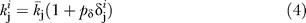
for *i* = 1, 2, …, *n*_c_ − 1, *n*_c_ and *j* = 1, 2, …, 13, 14, where 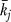 is the nominal value of *k_j_* (given in Figure 1), *p_δ_* is the level of perturbation, i.e., 0.05, 0.1, or 0.2, and *δ ^i^_j_* is a uniformly distributed random number between −1 and +1. The initial condition for internal cAMP is randomly sampled from the following:

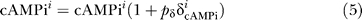
for *i* = 1, 2, …, *n*_c_ − 1, *n*_c_, where cAMPi *^i^* is the nominal initial value of cAMPi for the *i*-th cell and *δ ^i^*_cAMPi_ is a uniformly distributed random number between −1 and +1. The sampling for the other molecules is defined similarly. The nominal initial value for each molecule is given by [5] as: ACA = 7290, PKA = 7100, ERK2 = 2500, RegA = 3000, cAMPi = 4110, cAMPe = 1100, and CAR1 = 5960. Similarly, the cell volume is perturbed as follows:

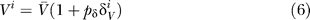
for *i* = 1, 2, …, *n*_c_ − 1, *n*_c_, where 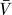 = 3.672 × 10^−14^ *l* and is a uniformly distributed random number between −1 and 1.

Although some of the nominal parameter values in the model were derived from (inherently noisy) biological data, others were tuned to values which generated the required oscillatory behaviour.

Thus, we have very little a priori information on the likely distributions of the parameters as a result of environmental variations and modelling uncertainty. In such cases, the uniform distribution is the standard choice for the type of statistical robustness analysis performed in this paper. Indeed, this is the approach adopted in several previous studies of robustness in biomolecular networks, [31,32]. Even if the true distribution were in fact a normal distribution, unless the variance is very small the robustness analysis results obtained with the uniform distribution would not be significantly different.

The simulations for the deterministic model and the stochastic model are performed using the Runge-Kutta 5^th^-order adaptive algorithm and the τ-leap complex algorithm [33], with the maximum allowed relative errors 1 × 10^−4^ and 5 × 10^−5^ respectively, which are implemented in the software Dizzy, version 1.11.4 [34].

### Calculating period and amplitude distributions.

From the simulations, the time series of the internal cAMP concentration is obtained with a sampling interval of 0.01 min from 0 to 200 min. Taking the Fourier transform using the fast Fourier transform command in MATLAB [35], the maximum peak amplitude is checked and the period is calculated from the corresponding peak frequency. If the neighbourhood amplitudes around the peak amplitude are greater than 70% of the peak amplitude, i.e., the signal with the peak amplitude is not a significantly dominant one, then the signal is considered to be non-oscillatory.

## Abbreviations

cAMP: adenosine 3′, 5′-cyclic monophosphate
